# Real-time imaging of photosynthetic oxygen evolution from spinach using LSI-based biosensor

**DOI:** 10.1038/s41598-019-48561-y

**Published:** 2019-08-22

**Authors:** Shigenobu Kasai, Yamato Sugiura, Ankush Prasad, Kumi Y. Inoue, Teruya Sato, Tomohiro Honmo, Aditya Kumar, Pavel Pospíšil, Kosuke Ino, Yuka Hashi, Yoko Furubayashi, Masahki Matsudaira, Atsushi Suda, Ryota Kunikata, Tomokazu Matsue

**Affiliations:** 1 Graduate Department of Environmental Information Engineering, Tohoku Institute of Technology, Sendai, Japan; 2 Biomedical Engineering Research Center, Tohoku Institute of Technology, Sendai, Japan; 3 Department of Biophysics, Centre of the Region Haná for Biotechnological and Agricultural Research, Faculty of Science, Palacký University, Olomouc, Czech Republic; 4 Graduate School of Environmental Studies, Tohoku University, Sendai, Japan; 5 Graduate School of Engineering, Tohoku University, Aoba-ku, Sendai, Japan; 6 Japan Aviation Electronics Industry, Limited, Tokyo, Japan

**Keywords:** Biophysical methods, Imaging

## Abstract

The light-driven splitting of water to oxygen (O_2_) is catalyzed by a protein-bound tetra-manganese penta-oxygen calcium (Mn_4_O_5_Ca) cluster in Photosystem II. In the current study, we used a large-scale integration (LSI)-based amperometric sensor array system, designated Bio-LSI, to perform two-dimensional imaging of light-induced O_2_ evolution from spinach leaves. The employed Bio-LSI chip consists of 400 sensor electrodes with a pitch of 250 μm for fast electrochemical imaging. Spinach leaves were illuminated to varying intensities of white light (400–700 nm) which induced oxygen evolution and subsequent electrochemical images were collected using the Bio-LSI chip. Bio-LSI images clearly showed the dose-dependent effects of the light-induced oxygen release from spinach leaves which was then significantly suppressed in the presence of urea-type herbicide 3-(3,4-dichlorophenyl)−1,1-dimethylurea (DCMU). Our results clearly suggest that light-induced oxygen evolution can be monitored using the chip and suggesting that the Bio-LSI is a promising tool for real-time imaging. To the best of our knowledge, this report is the first to describe electrochemical imaging of light-induced O_2_ evolution using LSI-based amperometric sensors in plants.

## Introduction

Molecular oxygen (O_2_) during the evolution is known to be introduced into the environment about ~2.7 billion years ago by the O_2_ evolving photosynthetic organisms^1^. In the photosynthetic organisms, chloroplast and mitochondria are the main organelles responsible for the production and consumption of O_2_, respectively^2^. Photosynthetic water oxidation to O_2_ is catalyzed by a tetra-manganese penta-oxygen calcium (Mn_4_O_5_Ca) cluster bound to the proteins of photosystem II (PSII)^3–6^. Several techniques have been used in the past to measure O_2_ evolution in plants^7–11^. Each method utilized for detection and estimation of O_2_ has some strengths and limitations. The electrochemical method has been used for detection of O_2_ concentration including the Clark-type and Joloit-type electrode^12–14^. Quenching based O_2_ sensors which are prepared by embedding the fluorophore in O_2_ permeable polymer matrix [Polyvinyl chloride (PVC) or silicone] have an advantage as they are generally usable in both liquid and gaseous phase^9^. As quenching-based oxygen sensors, Ru(II) α-diimine complexes (15) and Pt(II)/Pd(II) porphyrin systems are among the most studied^15–18^. In addition to the above techniques, EPR oximetry, mass spectrometry, photoacoustic spectroscopy  cannot be easily used in plant systems and therefore not widely employed in photosynthetic research. The electrochemical technique has an advantage of direct detection of O_2_ without labels and additional reagents. As an example, electrochemical O_2_ analyzers based on the reduction of O_2_ at a negatively polarized platinum (Pt; electrode diameter: 25–75 μm) electrode have been demonstrated^19,20^. An ultra-microelectrode (diameter: 1.5 μm) was also introduced and inserted into a single cell to determine intercellular reduction current for O_2_^19,20^. More recently, scanning electrochemical microscopy (SECM) has been used for the imaging of O_2_ evolution during photosynthesis around single living cells^21^. SECM provides in addition to topographic information’s, more detailed information (temporal) on the cellular activity such as respiration etc.^22–24^.

In the present study, we used our recently developed LSI-based amperometric sensor array system referred to as Bio-LSI^25,26^ for two-dimensional imaging of light-induced O_2_ evolution from spinach leaves. The Bio-LSI comprised of a 10.4 mm × 10.4 mm complementary metal-oxide semiconductor (CMOS) sensor chip with 20 × 20 unit cells, an external circuit box, a control unit for data acquisition, and a DC power box. Each unit cell of the chip consists of an operational amplifier with a switched-capacitor type I–V converter for in-pixel signal amplification, which realizes a fast acquisition of electrochemical images with high sensitivity. On the Bio-LSI chip, the spinach leaves were illuminated with white light to induce O_2_ evolution and simultaneous imaging was performed. To the best of our knowledge, this is the first study to describe real-time electrochemical imaging of light-induced oxygen release from a photosynthetic organism using LSI-based amperometric sensors.

## Material and Methods

### Sample and chemical reagents

Spinach (*Spinacia oleracea)* leaves were purchased from the local market and washed twice with deionized water. The leaves were stored in dark for two hours before measurement to avoid any kind of light interference during the measurement. For each measurement, a fresh spinach leaf of the approximately same age and size were chosen. The experiments were performed under the dark condition and at room temperature (25 °C). 3-(3, 4-dichlorophenyl)-1, 1-dimethylurea (DCMU) ≥98% was purchased from Wako Pure Chemicals Industries, Ltd. (Osaka, Japan).

### Fabrication of Bio-LSI chip and measurement setup

**Figure 1:**
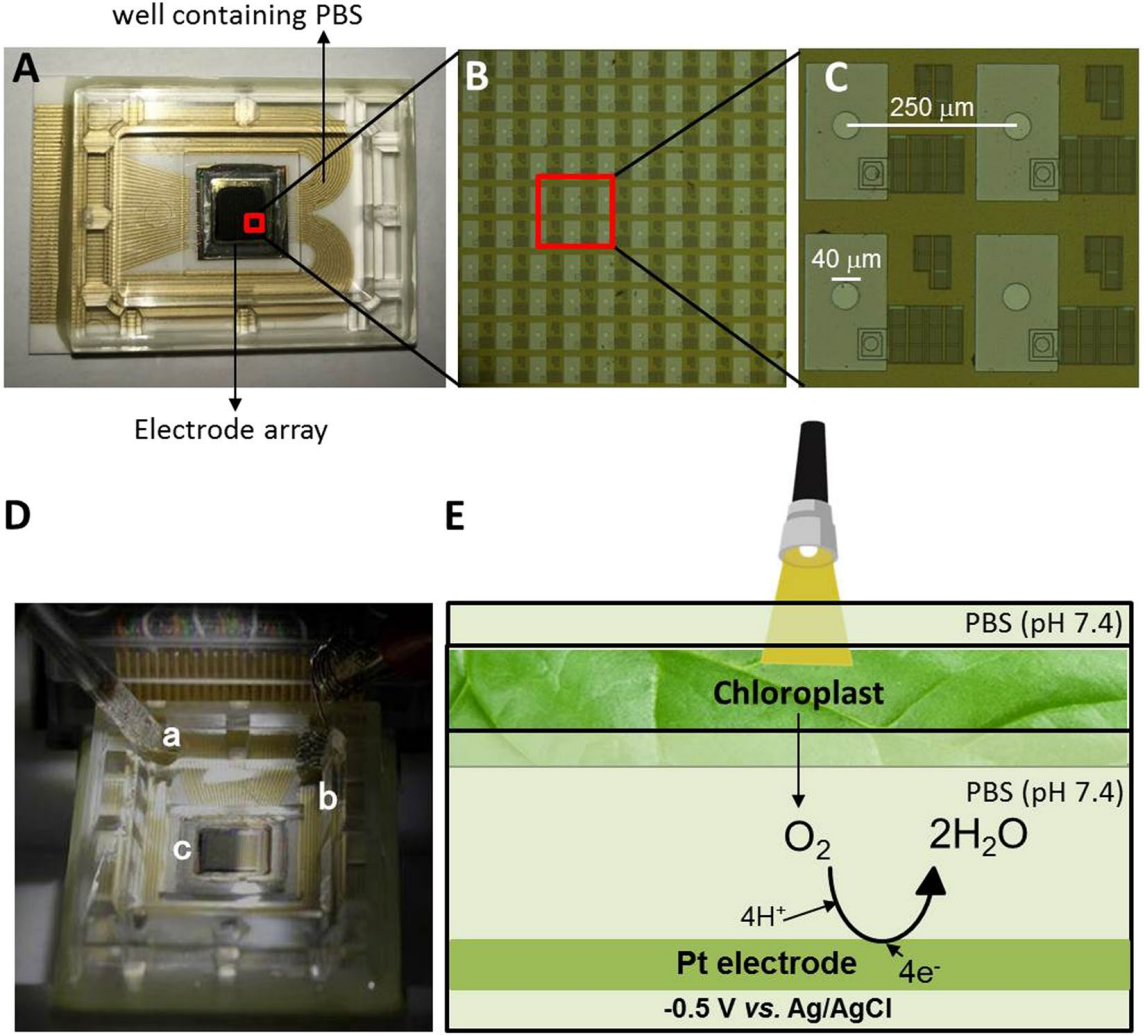
The photograph of the Bio-LSI chip (**A**), magnified images of Bio-LSI chip showing sensor points (**B**) and sensor electrodes (**C**). The diameter of each electrode is 40 μm and the distances between the electrodes are 250 μm. The measurement setup of electrode cell (**D**) with an RE (a) and a CE (b) placed in the same well as WEs (c). Schematic illustration of the measurement system (**E**) for the photosynthetic activity in plant tissues by detecting the O_2_ as reduction current on the WEs of Bio-LSI.

The detailed fabrication process for the sensor chip has been described in our previous study^27^. In summary, it consists of 400 working electrodes arranged in an array of 20 × 20 electrodes as represented in Fig. 1(A–C). The overall measurement setup consists of a reference electrode (RE): Ag/AgCl; a counter electrode (CE): a platinum (Pt) wire and 400 working electrodes (WEs) on the Bio-LSI chip (Fig. 1D). The WE is a Pt electrode each with a diameter of 40 μm. The RE and CE were inserted from the top as represented in Fig. 1D while the leaf excised sample rests above the WEs. The distance between the leaf sample and the Bio-LSI chip was less than ~1 mm due to the uneven surface area of the leaf. Some parts of the sample contacted to the sensing area. The ventral side of the leaf known to possess the stomata was placed on the electrode part for the purpose of acquiring the image. In the well positioned above the Bio-LSI chip, a phosphate buffer saline (PBS) (40 mM) at pH 7.4 was accommodated. The setup was established precisely using a stereoscopic microscope (Leica S8 APO).

### Deoxygenation

To avoid any interference of dissolved oxygen (DO) in the system containing PBS (without leaves), we conducted deoxygenation in the measurement well by applying a potential of −0.5 V *vs*. Ag/AgCl to the WEs before measurements. Reaction on the Pt WEs during the deoxygenation was as follows.

$${{\rm{O}}}_{{\rm{2}}}+{{\rm{4H}}}^{+}+{{\rm{4e}}}^{-}\to {{\rm{2H}}}_{{\rm{2}}}{\rm{O}}$$

**Figure 2:**
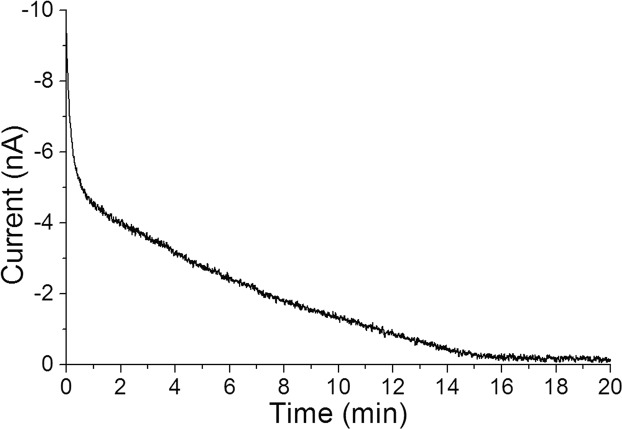
Time course of the deoxygenation. The DO reduction current on a WE of a Bio-LSI chip was monitored during the pretreatment for deoxygenation of the well. A voltage of −0.5 V vs. Ag/AgCl was applied to 400 WEs for 20 min.

The monitored current generated by the above reaction was gradually decreased from −8 nA to ~−0.2 nA in the time span of 20 min (Fig. 2). The remaining current was derived from diffusion of fresh oxygen from the atmosphere. From this result, we consider that the deoxygenation was completed in about 20 min.

### Real-time imaging and current monitoring of oxygen evolution during photosynthesis using Bio-LSI

For the real-time imaging of O_2_ evolution during the light-induced photosynthetic process, PBS was filled in the well containing WEs (Fig. 1A). Prior to this, spinach leaf in the dimension 5 mm × 2 mm was positioned on the WEs. A potential of −0.5 V *vs*. Ag/AgCl was applied to the WEs until the reduction current of DO was stabilized to ~0 nA. This was followed by two-dimensional imaging of O_2_ evolution from the spinach leaf by continuous application of −0.5 V *vs*. Ag/AgCl to the WEs. The light exposure was achieved using a KL300 LED light source (λ = 400–800 nm) connected with a light guide (Schott AG, Hattenbergstrasse 10, Mainz, Germany) with light intensities of 0 klx, 3 klx (40 μmol photons s^−1^ m^−2^), 20 klx (260 μmol photons s^−1^ m^−2^) and 30 klx (400 μmol photons s^−1^ m^−2^). The intensities of light in the current study were chosen based on the data provided by the Osaka Science Museum, Osaka, Japan. The light intensities used in our study reflect the situation close to field conditions. As a negative control, we performed the same experiments in the absence of spinach leaf. For time course monitoring of the reduction current, current data from a single randomly selected electrode (indicated by a red open square) from the electrode sensor array system was chosen. We also examined the effect of a photosynthetic inhibitor on the oxygen evolution by addition of DCMU prior to start (Supplementary Data 1) and during measurements. All images presented in the manuscript are representative figures of at least 3 measurements.

## Results

### Real-time imaging of oxygen evolution during photosynthesis with different intensities of illumination

**Figure 3:**
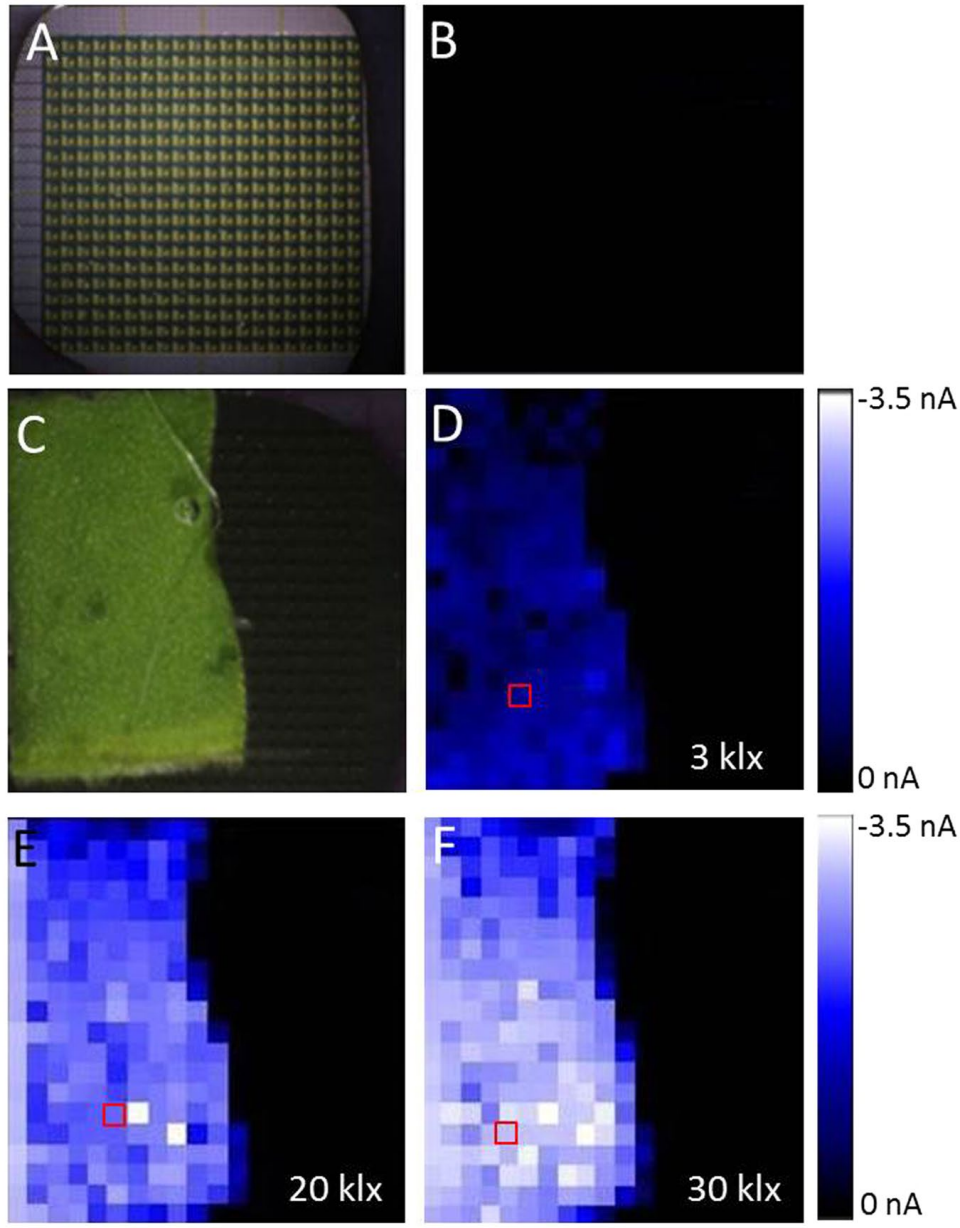
Photograph of 400 WEs on a Bio-LSI (**A**) and an image of O_2_ distribution based on the reduction current of O_2_ measured in the absence of spinach leaf (**B**). A photograph showing the arrangement of spinach leaf on the electrode surface (**C**) and images of O_2_ evolution after 5 min of light exposure from the spinach illuminated with 3 klx (**D**), 20 klx (**E**) and 30 klx (**F**).

Real-time imaging of O_2_ evolution from a spinach leaf was performed under the different illumination intensities (3 klx, 20 klx, and 30 klx). The spatial distribution of O_2_ was monitored as O_2_ reduction current measured with the Bio-LSI chip applied with −0.5 V *vs*. Ag/AgCl. Figure 3A shows the photograph of 400 WEs on a Bio-LSI and Fig. 3B shows a two-dimensional image of O_2_ reduction current measured without any illumination in the absence of spinach leaf. Figure 3C shows the arrangement of spinach leaf on the electrode surface of the Bio-LSI chip during the measurements. Figures 3D–F are the two-dimensional images for O_2_ reduction current measured at the illumination of 3 klx, 20 klx and 30 klx, respectively. Supplementary Data 2 shows additional set of data on two-dimensional imaging of O_2_ reduction current measured at the illumination of 3 klx, 10klx, 20 klx and 30 klx. The electrodes close with the leaf surface showed a change in reduction current in the range of −0.5 nA to ~−3.5 nA, while the electrodes far from the leaf did not show any significant changes in the reduction current. With the increase of light intensities, the change in O_2_ reduction current was increased. Supplementary Data 3 (video 1) shows the change in O_2_ reduction current in real-time during the turning on/off the light  at 30 klx. As a control experiment, real-time imaging of O_2_ evolution from the dorsal side of the spinach leaf bearing no stomata was measured (data not shown). No significant change in O_2_ reduction current was observed which is believed to be due to non-feasible long-distance diffusion of oxygen.

### Time course of oxygen evolution during photosynthesis at different intensities of illumination

**Figure 4:**
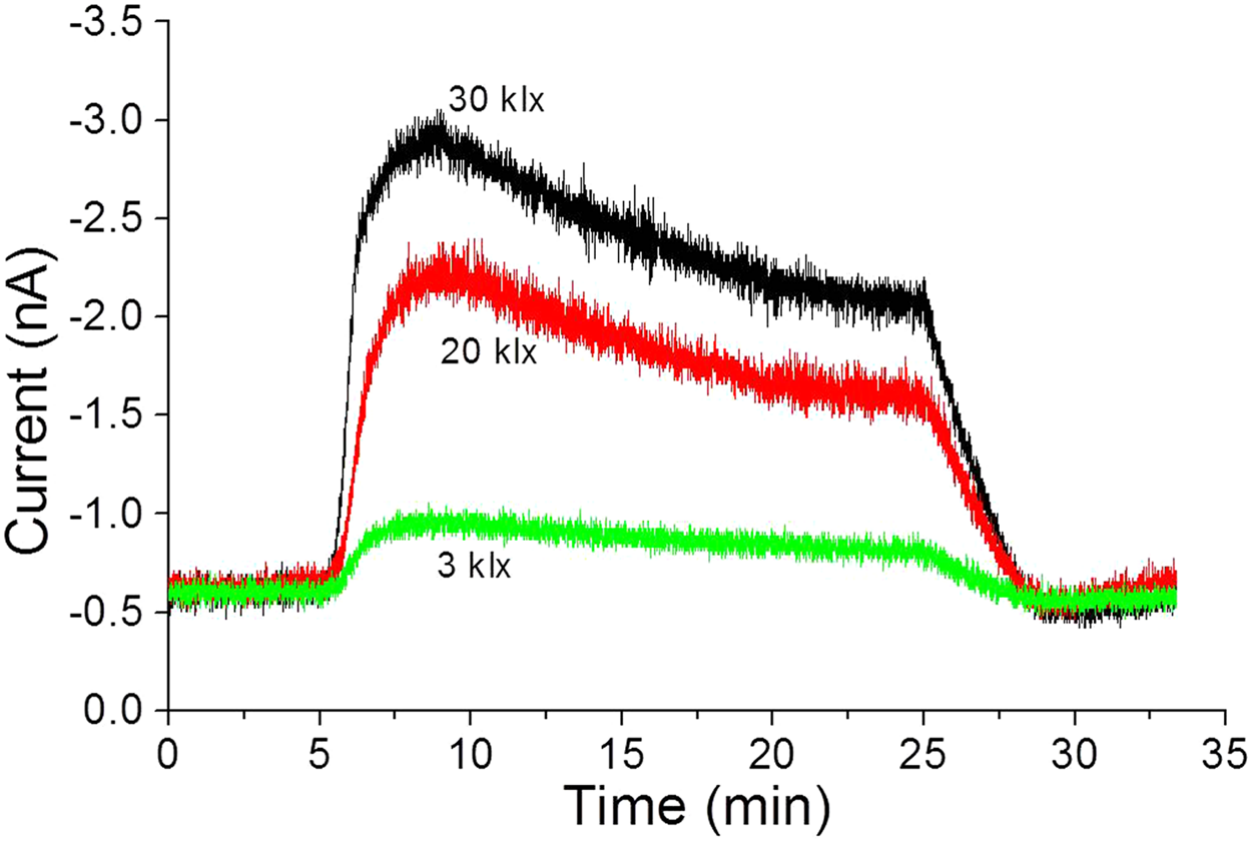
Changes in O_2_ reduction current of a single randomly selected electrode (indicated by a red open square in Fig. 3) measured with a spinach leaves illuminated by 3 klx (green trace), 20 klx (red trace) and 30 klx (black trace).

The time course of O_2_ evolution from spinach leaves during illumination with different intensities was compared. The changes in O_2_ reduction current monitored at a single electrode of the electrode array system are shown in Fig. 4. Exposure to white light at 3 klx leads to change in O_2_ reduction current by −1 nA which was significantly increased to −2.3 nA and −3.0 nA with exposure to light intensities of 20 klx and 30 klx, respectively. The O_2_ evolution depending on the illumination intensity was clearly monitored by the Bio-LSI system. With the turning off the light, the oxygen reduction current showed a sharp decrease to a value equivalent to the value observed during first 5 min (no illumination). It can also be seen that the reduction current value began to gradually decrease after 5 min of illumination (Fig. 4, red and black trace). This may be because of consumption of O_2_ due to continuous application of a potential to the electrode, O_2_ reduction by PSII and limited O_2_ production by the plant tissue.

**Figure 5:**
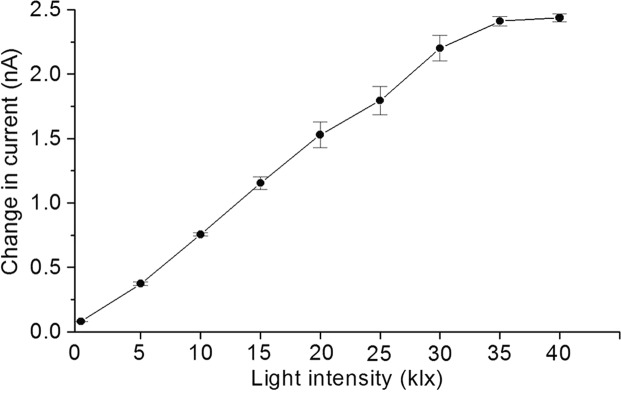
Change in O_2_ reduction current (*ΔI*) showing the difference between the background current and value of oxygen reduction current (moving average) measured using Bio-LSI based on the experimental conditions described in Fig. 4. The change in O_2_ reduction current (*ΔI*, in nA) was measured during the illumination at intensity range of 0–40 klx.

For a further understanding of the behavior of O_2_ evolution during illumination, the change in O_2_ reduction current (*ΔI*) from the current just before the turning on of the light at different period of illumination were plotted as averages of 3 randomly selected electrodes under the leaf with error bars indicating standard deviations. Figure 5 shows the *ΔI* at light intensities in the range of 0–40 klx. The O_2_ evolution reflected by a change in reduction current was almost proportional to light intensity up to 30 klx; however, the response was saturated when the light intensity exceeded 35 klx. The saturation intensity and oxygen-evolution rate are important factors describing the photosynthetic capability of plant leaves. The above results indicate that amperometric measurement using Bio-LSI is applicable to screen the photosynthetic capability of the leaves including the oxygen constraining process of carbon fixation.

**Figure 6:**
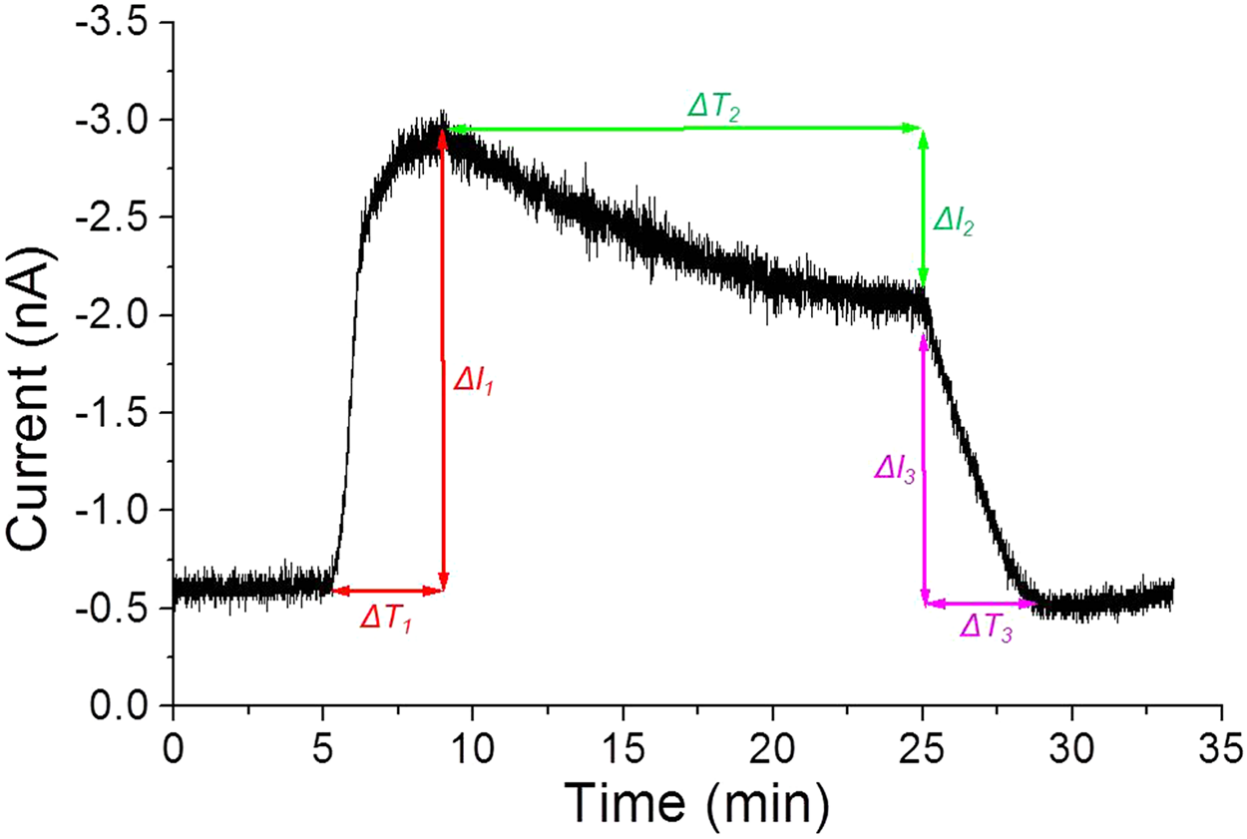
Details showing parameters utilized for calculation of oxygen evolution. Changes in O_2_ reduction current of a single randomly selected electrode (indicated by a red open square in Fig. 3) measured with spinach leaves illuminated at 30 klx. V, *ΔI* and *ΔT* represents the concentration of evolved O_2_, change in O_2_ reduction current and time duration, respectively. The parameters were considered for calculations of oxygen evolution as presented in Table 1.

In order to estimate the molecules of O_2_ evolved, the background reduction current value of the measurement solution and the DO concentration (estimated to be about 253 μM in the case of an electrode having a measurement solution temperature of 25 °C and a radius of 40μm) were taken into account. The oxygen concentration was recalculated and has been presented in Table 1 based on parameters shows in Fig. 6.

**Table 1:** Oxygen evolution (in nmol) calculated using standard bulk dissolved oxygen concentration measured in the case of a Pt electrode (diameter 40 μm) having a measurement solution (PBS) and temperature of 25 °C.

	ΔI_1 (nA)_	ΔT_1 (sec)_	V_1 (nmol/sec)_	ΔI_2 (nA)_	ΔT_2 (sec)_	V_2 (nmol/sec)_	ΔI_3 (nA)_	ΔT_3 (sec)_	V_3 (nmol/sec)_
3klx	0.3	200	47	0.1	880	4	0.2	240	26
20klx	1.6	240	211	0.6	840	23	1.0	240	132
30klx	2.3	240	303	0.8	840	30	1.5	240	198

The oxygen evolution (V, in nmol) was calculated during the illumination at the intensity range of 3 klx, 20 klx and 30 klx.

### Real-time imaging of oxygen reduction current with photosynthetic inhibition

**Figure 7:**
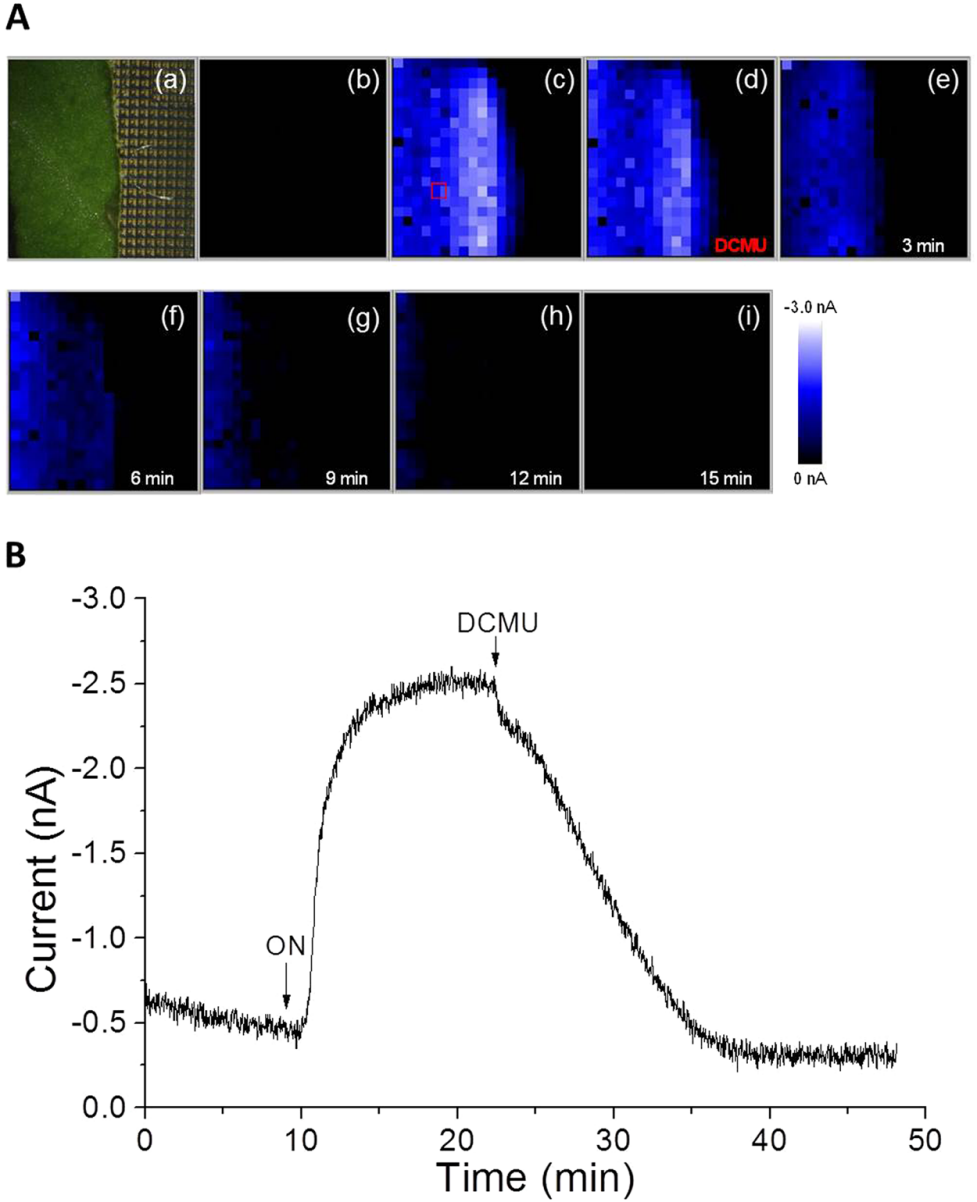
(**A**) Photograph showing the arrangement of spinach leaf on the Bio-LSI chip (a), real-time imaging of O_2_ reduction current without illumination (b) and with illumination at 30 klx (c). DCMU (5 μM) was added after 13 min from the start of illumination (30 klx) and subsequent images were obtained at 0 min (d), 3 min (e), 6 min (f), 9 min (g), 12 min (h) and 15 min (i). (**B**) The change in reduction current on an electrode indicated by a red open square in Fig. 7A(c). The arrow indicates the time of turning on the light (at 10 min) and subsequent addition of DCMU (at 23 min).

**Figure 8:**
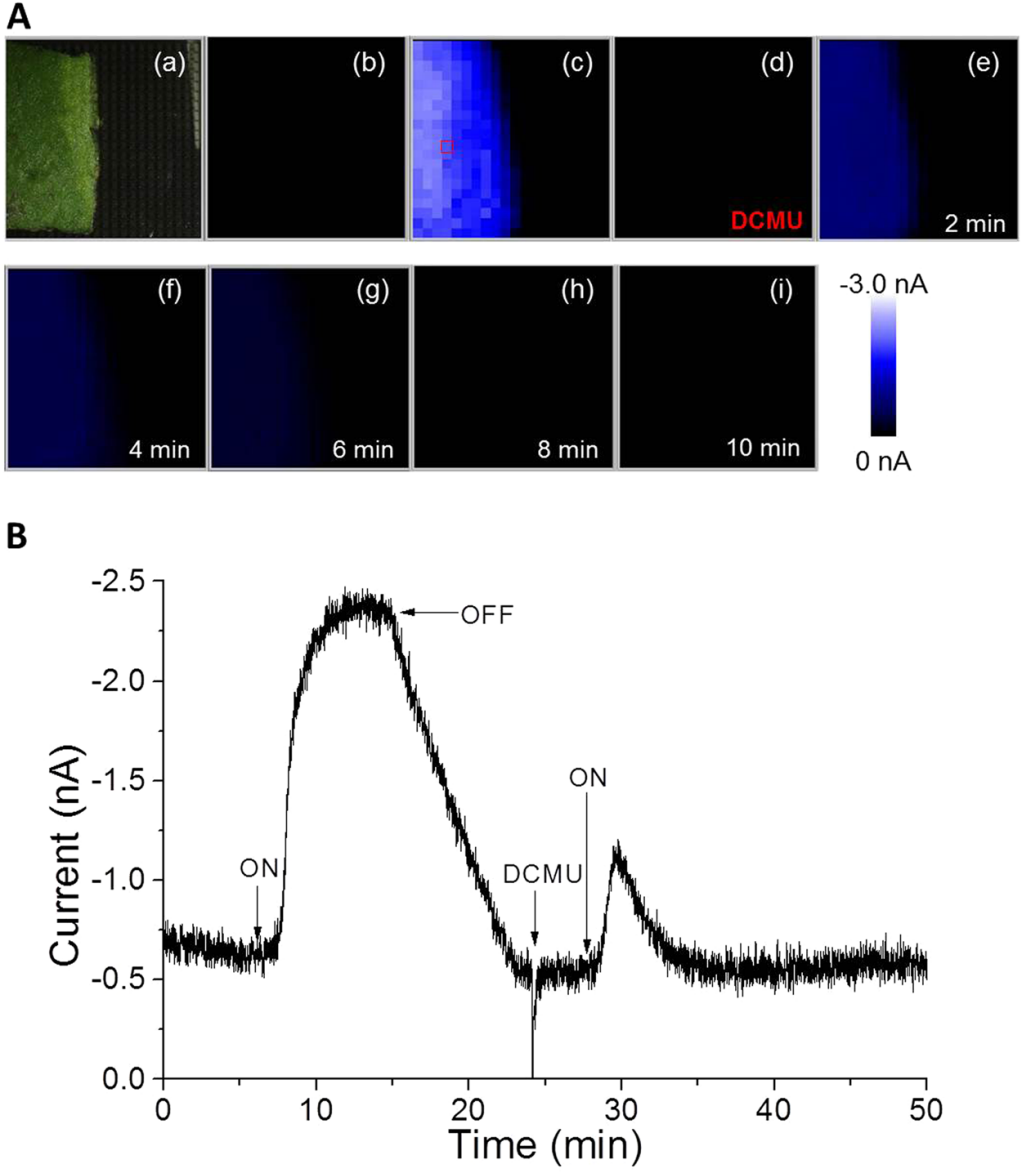
(**A**) Photograph showing the arrangement of spinach leaf on the Bio-LSI chip (a), real-time imaging of O_2_ reduction current without (b) and with illumination at 30klx (c). The illumination was turned off 10 min after the start of illumination and DCMU (5 μM) was added 10 min after turning off the light. Then, the illumination was turned on again after the addition of DCMU and subsequent images were taken at 0 min (d), 2 min (e), 4 min (f), 6 min (g), 8 min (h), 10 min (i) after turning on the light (at 28 min). (**B**) The change in reduction current measured on a single randomly selected electrode (indicated by a red open square in Fig. 8C).

Real-time imaging of O_2_ reduction current was performed in the presence of photosynthetic inhibitor of electron transport in PSII DCMU (5 μM) to confirm the origin of the reduction current. Figure 7A shows the arrangement of spinach leaf on the Bio-LSI chip (a), an image of O_2_ reduction current before illumination (b) and an image during illumination (30 klx) (c). To test the effect of photosynthetic inhibitor, DCMU was exogenously added during the illumination (d) and subsequent images were taken after every 3 min for a time span of 15 min (e-i). At the initial 5 min of DCMU addition, partial inhibition of oxygen evolution was observed and then, the inhibition was increased with time leading to complete inhibition after 15 min (i). Figure 7B shows the time course of the O_2_ reduction current changes on one electrode [indicated by a red open square in Fig. 7A(c)]. In addition to the above measurement, light-induced O_2_ evolution was also tested after the suppression by DCMU (Fig. 8). Figure 8A shows the arrangement of spinach leaf on the Bio-LSI chip (a), an image of O_2_ reduction current without illumination (b), and during illumination (30 klx) (c). After a suppression in O_2_ evolution by addition of 5 μM DCMU, illumination was performed again, and subsequent images were taken every 2 min (e-i). Only a slight increase in oxygen reduction current was observed after the illumination which was found to rapidly decrease within 2 min. Figure 8B shows the changes in O_2_ reduction current showing similar effects. With the first turning on of light illumination at 5 min in the absence of DCMU, the O_2_ reduction current increased to reach to −2.5 nA. With the turning off the light, the current decreased to −0.5 nA within 5 min. With the addition of DCMU at this point and at a lapse of 5 min, the light source was turned on again. The small increase of the O_2_ reduction current was observed but the peak of the current was reduced by about 1/2 and quickly decreased due to the inhibitory effect of DCMU on the O_2_ evolution activity of the photosynthesis. These results indicate the potential of the Bio-LSI to be used for the monitoring the O_2_ evolution activity as well as the impact of the environmental stresses to the tissues.

## Discussion

In this study, we demonstrated real-time monitoring of O_2_ evolution from a spinach leaf with light illumination by a change in O_2_ reduction current using an LSI-based amperometric sensor array system called Bio-LSI. With the increase of the light intensities from 0 klx to 30 klx, the reduction current reflecting oxygen evolution from the leaf was increased almost proportional to light intensity while the electrodes far from the leaf did not show any significant changes in the reduction current (Figs 3, 7A, 8A). With the turning off the light, the oxygen reduction current showed a sharp decrease to a value equivalent to first 5 min. In addition, we observed a decrease of O_2_ reduction current with applying 5 μM of photosynthetic inhibitor DCMU to the illuminated leaf, showing that we detected the photosynthetic activity of plant tissue (Fig. 7B). This technique can be broadly applied to investigate the effect of chemical and physical stimulation on the photosynthetic organism including the impact of the environmental stresses to the plant tissues.

Several conventional methods including polarography, EPR oximetry, mass spectrometry, photoacoustic spectroscopy and galvanic sensors have been used to measure photosynthetic oxygen evolution over the past years; however, most of them can only offer mean values which lack spatial resolution and are therefore blind to any compartmentation within the sample^28^. The imaging methods for oxygen consumption, oxygen evolution and subsequent reactive oxygen species characterization which were used during the past decades in photosynthetic research were mainly the fluorescence-based measurements. Fluorescence measurements offer wide applicability; however undesired fluctuation by quenching/emission from other materials, shielding due to the solution (turbidity of the medium) and need for probe labelling has to be considered. As far as probes labelling is concerned, there are several issues related which includes but not limited to fluorescent probes cross-reactivity, the existence of endogenous fluorochromes, excitation and emission wavelengths overlap, probe uptake, toxicity-either of the fluorescent compounds and/or solvent etc. In the current study, the Bio-LSI chip has been presented for the real-time imaging of photosynthetic oxygen evolution from spinach. Through the modification of electrodes, the use of the device can be extended for detection of other chemical species/reactive species. Bio-LSI chip has an advantage considering the simplicity and easy handling of the measurement setup with no interferences from exogenous probes/chemicals and wider applicability. It is possible to detect oxygen production rate with higher sensitivity [in the range of nmol/sec] and from larger surface area. Thus, it offers multi-point biosensing with higher sensitivity. The current system is non-invasive and future development w.r.t sensing points can enhance spatial and temporal resolution considerably. The Bio-LSI being equipped with CMOS sensor chip and operational amplifier within each unit cell not only makes it compactly designed and easy to handle but also offer in-pixel signal amplification, fast acquisition and high sensitivity which makes its a promising tool in photosynthetic research and more broadly in investigating different aspect in plant science research.

Some technical limitations are however associated with Bio-LSI especially in the context of temporal measurements. The data resulting from 400 electrodes at 100ms interval will be enormous and thus choosing to follow 1 electrode/up to few electrodes for plotting the temporal distribution of changes in oxygen reduction current presented as Figs 4, 7B, 8B are more feasible. Spatial resolution can also be affected to some extent since the distance between the leaf sample and the Bio-LSI chip at different electrode positions can slightly vary due to the uneven surface area of the leaf or any other biological sample. Since some part of the sensing area can be closer than other (mostly in case of solid samples), the change of the oxygen concentration can be subject to some errors. Thus, the diffusion of oxygen in the medium has to be taken into account and should not be completely neglected. Although there is a short delay due to the diffusion of molecules from targets to sensors in electrochemical imaging, “real-time” is generally used^29^.

## Supplementary information

Supplementary dataset 1–2

Supplementary dataset 3
