# Supplementary material for: Real-time imaging of photosynthetic oxygen evolution from spinach using LSI-based biosensor
Source: Sci Rep. 2019 Aug 22;9:12234. doi: 10.1038/s41598-019-48561-y (PMC6706413; doi:10.1038/s41598-019-48561-y)
Supplement: Supplementary file 1 — Supplementary dataset 1–2 [file 41598_2019_48561_MOESM1_ESM.pdf]

Real-time imaging of photosynthetic oxygen evolution from spinach using LSI-based biosensor

Shigenobu Kasai[1,2]*, Yamato Sugiura[1], Ankush Prasad[3#], Kumi Y Inoue[4], Teruya Sato[1], Tomohiro Honmo[1], Aditya Kumar[3], Pavel Pospíšil[3], Kosuke Ino[5], Yuka Hashi[4], Yoko Furubayashi[4], Masahki Matsudaira[4], Atsushi Suda[6], Ryota Kunikata[6] and Tomokazu Matsue[4,5]

[1]Graduate Department of Environmental Information Engineering, Tohoku Institute of Technology, Sendai, Japan
[2]Biomedical Engineering Research Center, Tohoku Institute of Technology, Sendai, Japan
[3]Department of Biophysics, Centre of the Region Haná for Biotechnological and Agricultural Research, Faculty of Science, Palacký University, Olomouc, Czech Republic
[4]Graduate School of Environmental Studies, Tohoku University, Sendai, Japan
[5] Graduate School of Engineering, Tohoku University, Aoba-ku, Sendai, Japan
[6]Japan Aviation Electronics Industry, Limited, Tokyo, Japan

***Corresponding author:**

S. Kasai, Graduate Department of Environmental Information Engineering, Tohoku Institute of Technology, Sendai, Japan.
Tel.: +81-22-305-3970; Fax: +81-22-305-3970
E-mail address: kasai@tohtech.ac.jp

#Co-Corresponding author:

E-mail address: prasad.ankush@gmail.com

Supplementary Fig S1

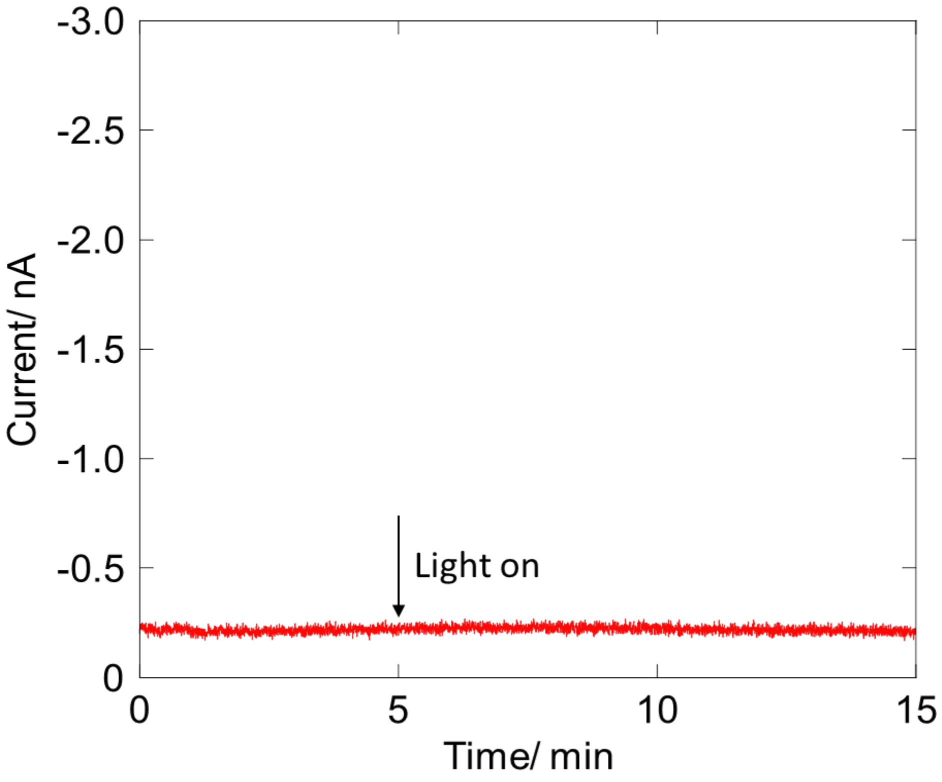

Figure S1: Real-time monitoring of oxygen reduction current in system containing spinach leaf in the presence of DCMU (5mM). The spinach leaf was illuminated at 3klx.

Supplementary Fig S2

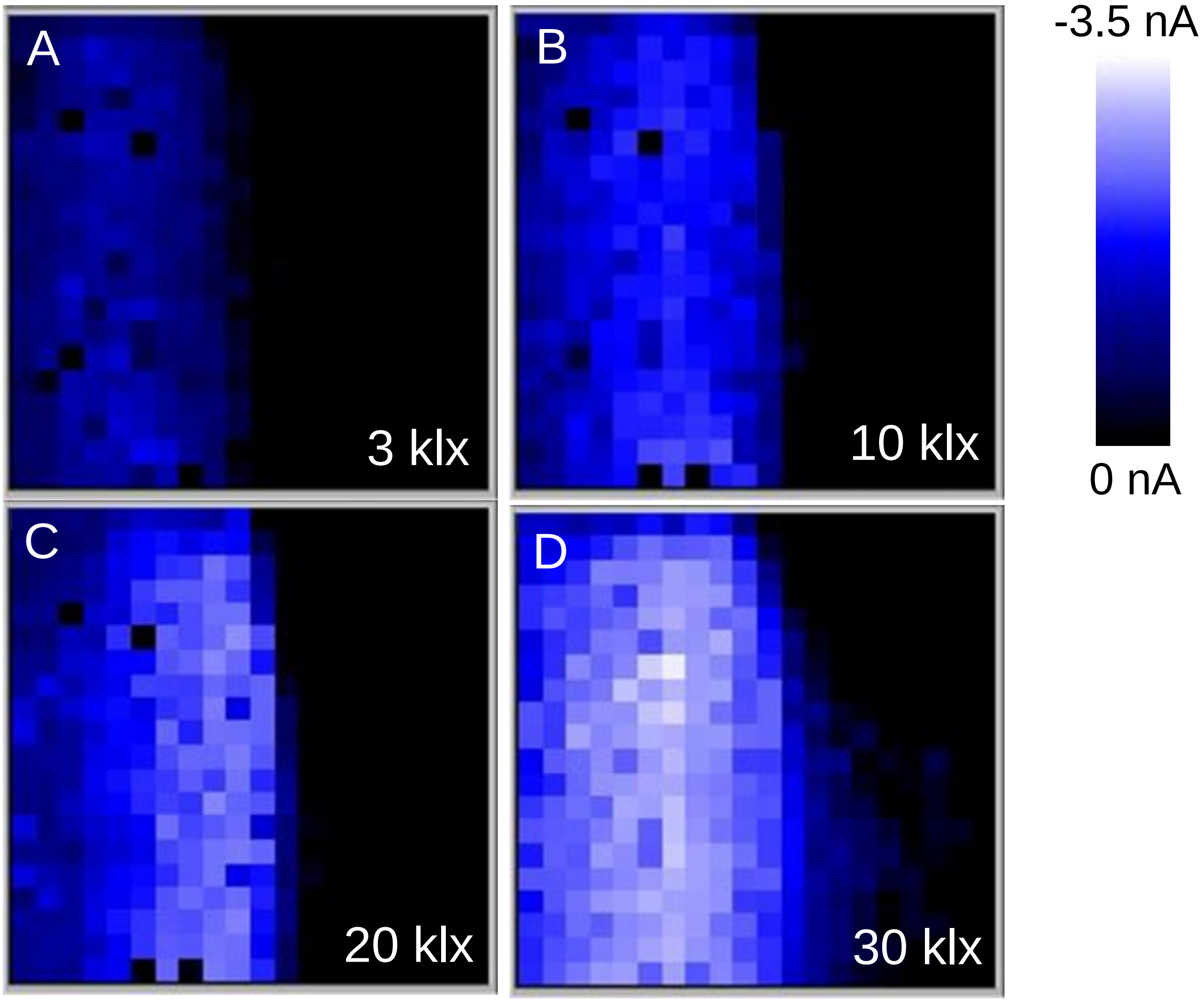

Figure S2: Biological replicate on O_2 evolution at different light intensities. Images of O_2 evolution after 5 min of light exposure from the spinach illuminated with 3klx (A), 10klx (B), 20klx (C) and 30klx (D).
